# TAK-242 alleviates diabetic cardiomyopathy via inhibiting pyroptosis and TLR4/CaMKII/NLRP3 pathway

**DOI:** 10.1515/biol-2022-0957

**Published:** 2024-09-10

**Authors:** Xiaolong Zhao, Jing Zhang, Feng Xu, Longqi Shang, Qingquan Liu, Chunjian Shen

**Affiliations:** School of Graduates, Dalian Medical University, Dalian, China; Medical Department, The Second Hospital of Dalian Medical University, Dalian City, China; Department of Nursing, The Second Affiliated Hospital of Shenyang Medical College, Shenyang City, China; Department of Cardiothoracic Surgery, The Fourth People’s Hospital of Shenyang, No. 20 Huanghe South Street, Shenyang, 110000, Liaoning, China

**Keywords:** CaMKII/NLRP3 pathway, diabetic cardiomyopathy, inflammasome, pyroptosis, TAK-242, TLR4

## Abstract

Diabetic cardiomyopathy (DCM) is identified as a progressive disease that may lead to irreparable heart failure. Toll-like receptor (TLR) signaling is believed to be implicated in the pathogenesis of DCM. This study intended to explore the potential impact of Toll-like receptor 4 (TLR4) on DCM *in vitro* and *in vivo*. Streptozotocin and HG medium were utilized to induce diabetes in animal and cell models, respectively. Selective TLR4 inhibitor TAK-242 and calcium/calmodulin-dependent protein kinase-II (CaMKII) inhibitor KN-93 were employed to explore the involvement of TLR4/CaMKII in DCM. TLR4 expression was increased in DCM hearts, while inhibition of TLR4 activation by TAK-242 improved cardiac function, attenuated heart hypertrophy, and fibrosis, as well as reduced oxidative stress and proinflammatory cytokine levels in rats, which were confirmed by Doppler echocardiography, hematoxylin and eosin staining, and Masson Trichome staining and specific enzyme-linked immunosorbent assay kits. Besides, the expression of hypertrophy-related molecules and oxidative stress damage were also inhibited by TAK-242. Furthermore, TAK-242 treatment reduced CaMKII phosphorylation accompanied by decreased expression of NOD-like pyrin domain-containing protein 3, gasdermin D (GSDMD), The N-terminal domain of Gasdermin D (GSDMD-N), apoptosis-associated speck-like protein containing a caspase-recruitment domain (ASC) and Caspase-1 both *in vivo* and *in vitro.* Similar positive impacts on HG-induced pyroptosis were also observed with KN-93 treatment, and this was achieved without affecting TLR4 expression. Collectively, our work suggested that TAK-242 demonstrated substantial benefits against DCM both *in vivo* and *in vitro*, potentially attributed to the suppression of the TLR4-mediated CaMKII/NLRP3 pathway activity.

## 1 Introduction

Diabetes mellitus (DM) is a chronic condition that poses a considerable public health burden, and its global prevalence is on the rise [1]. Diabetic cardiomyopathy (DCM), first proposed by Rubler, refers to a heart muscle condition that occurs in individuals with diabetes [2]. DCM is identified as impaired cardiac function in the absence of concurrent cardiovascular conditions, including coronary heart disease, systemic hypertension, valvar disease, or other structural abnormalities in the heart [3]. Notably, the progression of DCM typically starts with impaired diastolic function of the left ventricle and cardiac hypertrophy, eventually leading to systolic dysfunction and heart failure, accompanied by cardiomyocyte loss and fibrosis accumulation in the heart [4,5]. Despite numerous studies conducted on DCM, our understanding of its pathophysiology remains incomplete.

Pyroptosis has been proven to play a significant role in the progression of DCM. Elevated glucose levels can induce cell pyroptosis [6], and inhibiting pyroptosis has shown effectiveness in alleviating DCM symptoms [7,8]. The assembly of an inflammasome complex, along with caspase-1 and IL-1β, serves as the initiation of pyroptosis [9,10]. It is well understood that inflammatory factor tumor necrosis factor-alpha (TNF-α) is involved in DCM pathogenesis on account of its role in initiating several intracellular death signaling, including pyroptosis [11]. It is reported that TNF-α forms a complex by binding to TNF receptor 1, and the complex is validated to be involved in pyroptosis initiation [12,13]. Studying pyroptosis enhances our comprehension of the mechanisms contributing to DCM progression, which reveals novel targets for advancing treatment approaches.

Toll-like receptors (TLRs) are membrane-bound receptors that play a crucial role in innate immunity and are involved in the regulation of various signal transduction pathways [14]. TLR4, in particular, has been implicated as a key mediator of inflammation in diabetes and may contribute to the development of diabetic complications [15,16]. Multiple studies have evidenced that hyperglycemia activates TLR4 and induces inflammation in DCM [17]. Suppressing TLR4-associated pathways has been proven effective in mitigating DCM [18,19]. Additionally, a previous study has shown that TLR4 mediates cell pyroptosis through various mechanisms [20]. Hence, targeting TLR4 presents a promising approach for treating DCM by effectively inhibiting pyroptosis, which could offer valuable targets for the development of innovative therapeutic interventions.

Ca^2+^/calmodulin-dependent protein kinase (CaMK) family members are essential in a variety of pathophysiological functions, such as survival, proliferation, cell differentiation, and inflammatory disorders [21]. Ca^2+^/calmodulin-dependent protein kinase-II (CaMKII), one prominent member, acts as a multifunctional serine/threonine kinase that controls expressions of the downstream genes via activating transcription factors [22]. CaMKII is involved in initiating NOD-like pyrin domain-containing protein 3 (NLRP3) inflammasome signaling in cardiomyocytes, which triggers inflammation in non-ischemic heart disease [23]. Notably, CaMKII/NLRP3 signaling is involved in the process of pyroptosis, and targeting NLRP3 through the CaMKII/cyclic AMP response element binding protein axis has demonstrated significant alleviation symptoms in Alzheimer’s mouse models by inhibiting pyroptosis [24]. However, further research is required to elucidate the mechanistic role of CaMKII/NLRP3 in DCM.

Recent studies have highlighted the intervention methods targeting pyroptosis as a potential future direction for preventing and treating DCM [6,25], but the mechanism related to pyroptosis needs to be further studied. The previous study has reported that TLR4 activates the CaMKII Mst1/2-Rac axis by inducing calcium influx [26]. Consequently, our current investigation primarily focused on the effects and underlying mechanisms of TLR4 on HG-stimulated cardiac damage. Our findings demonstrated that inhibiting TLR4 suppressed pyroptosis, primarily through its inhibitory impact on the CaMKII/NLRP3 pathway. This study offers a new mechanistic understanding of the protective effects of TAK-242 in DCM.

## 2 Materials and methods

### 2.1 Animal model

The experiments were carried out on male Sprague Dawley (SD) rats, aged 8 weeks, weighing between 150 and 170 g. All protocols involving animal subjects adhered to the laboratory animal guidelines established by the US National Institutes of Health and received approval from the Animal Care and Use Committee of Dalian Medical University (no: CSE202301007). The experimental rats were maintained under specific pathogen-free conditions, with a conventional room setup and a 12:12-h light/dark cycle. Throughout the study, the animals were provided free access to food and water.

Male SD rats (aged 8 weeks, weighing between 150 and 170 g) were assigned at random into one of four groups following acclimatization for 1 week: control group (*n* = 8), DM group (*n* = 16), control+TAK-242 group (*n* = 8), and DM+TAK-242 group (*n* = 8). The rats in the DM and DM+TAK-242 groups were intraperitoneally administered with streptozotocin (STZ; 50 mg/kg; Sigma-Aldrich, USA) dissolved in citrate acid buffer dilution (Sigma-Aldrich, USA) for three consecutive days. Rats in the control and TAK-242 groups received the citrate buffer injection. Fasting blood glucose (FBG) levels were measured 1 week after STZ injection, and the rats with FBG levels above 16.7 mM were selected for further investigation [27]. The rats in the control+TAK-242 and DM+TAK-242 groups received daily intraperitoneal injections of TAK-242 (3 mg/kg, dissolved in DMSO) for seven consecutive days starting from 3 weeks after STZ administration. Both the control group and the DM group of rats were administered an equivalent volume of DMSO via intraperitoneal injection. Following 8 weeks of STZ injection, the DM group was further divided into two subgroups: the DM group (*n* = 8) and the DM+KN-93 group (*n* = 8). The DM+KN-93 group received intraperitoneal injections of KN-93 at a dosage of 10 mg/kg/2 days for 8 weeks, while rats in the other group received vehicle injection. Blood glucose levels were regularly monitored throughout the experiment. Following the completion of the echocardiogram (ECG), blood samples were collected, and cardiac tissues were obtained for cardiac histology and molecular biology studies. All animals were sedated utilizing 2% isoflurane administered by inhalation and underwent ECG testing to evaluate ST segment alterations. Echocardiography was performed using small-animal ultrasonography (Vinno Co., China) to assess various cardiac functions, including ejection fraction (EF), fractional shortening (FS), peak E-to-peak A ratio (E/A), and left ventricular internal dimension at systole (LVIDs).


**Ethical approval:** The research related to animals’ use has been complied with all the relevant national regulations and institutional policies for the care and use of animals.

### 2.2 Histopathology

Midventricular heart samples were fixed in a solution of 4% formalin, dehydrated with alcohol, embedded in paraffin, and cut into 5 μm sections using a microtome for histological examination. Hematoxylin and eosin staining was conducted on several transverse slices of paraffin-embedded samples to visualize general tissue morphology. Additional paraffin slices were stained with Masson trichrome to assess collagen deposition [28]. The sections were analyzed under a light microscope (Olympus, Japan), and photomicrographs of the tissue sections were captured with an Olympus microscope camera.

### 2.3 Cell culture

H9c2 cardiomyocytes, derived from rats, were obtained from the American Type Culture Collection. The cell line was cultivated in Dulbecco’s modified Eagle’s medium (DMEM; Cytiva, America) supplemented with 10% fetal bovine serum (Kangyuan Biotechnology, China) and 1% penicillin–streptomycin. The cells were maintained in a humidified incubator at 37°C with a 5% CO_2_ atmosphere. Cells between passages 3 and 8 were used for subsequent experiments. To simulate the hyperglycemic (HG) state of type-1 diabetes, a high glucose solution (30 mM glucose) was utilized. The cells were separated into two groups: normoglycemic (NG, 5.5 mM glucose) and HG. TAK-242 (1 mM) and KN-93 (5 μm) were added to determine the mechanism of action. According to the experimental design, the H9c2 cardiomyocytes were categorized into five groups based on the culture media and medications employed: NG (normal glucose), HG (high glucose), NG+TAK-242, HG+TAK-242, and HG+KN-93.

### 2.4 Cell Counting Kit-8 (CCK-8) assay

H9c2 cells were seeded onto a 96-well plate with a total amount of 1 × 10^4^ cells per well. Then, the cells were exposed to HG treatment, with or without TAK-242 (1 mM) and KN-93 (5 μM) for 48 h. The proliferation of cells was evaluated utilizing a CCK-8 Kit (Beyotime Biotechnology, China) in strict accordance with the manufacturer’s instructions. Following TAK-242 or KN-93 treatment, 10 μL of CCK-8 reagent was added, and then the cells were further incubated for 2 h at 37°C. The measurement of absorbance was taken at a wavelength of 450 nm.

### 2.5 Measurement of reactive oxygen species (ROS)

Intracellular ROS levels were evaluated using the 2′-7′-dichlorodihydrofluorescein diacetate (DCFH-DA) probe kit (Beyotime, China). After treating the cells with HG and/or TAK-242 for 24 h, the cells were washed three times with serum-free medium. Subsequently, the cells were incubated with DCFH-DA in the absence of light for 30 min. The fluorescence intensity was measured using a flow cytometer (ACEA, China) for quantitative analysis.

### 2.6 Terminal deoxynucleotidyl transferase-mediated deoxyuridine triphosphate-biotin nick end labeling (TUNEL) staining

The TUNEL staining agents (Beyotime, China) were used to stain the cell slides or tissue sections in accordance with the manufacturer’s instructions. After staining the nuclei with 4′,6-diamidino-2-phenylindole, the sections were sealed, and a fluorescent microscope (COIC, China) was used to capture the images.

### 2.7 Flow cytometry

Cell apoptosis was measured by employing flow cytometry. H9c2 cells were collected and stained with Annexin V-FITC and propidium iodide. The flow cytometer (ACEA, China) was utilized to evaluate the stained cells for the dead cells population following dual labeling for 15 min at room temperature and darkness [29].

### 2.8 Enzyme-linked immunosorbent assay (ELISA)

The levels of IL-1β and IL-18 in the cell culture supernatants or serum were determined using ELISA kits from Elabscience Biotechnology (China). The quantification was performed following the manufacturer’s instructions and guidelines provided with the kits [30].

### 2.9 Reverse transcriptase qPCR

The total RNA samples were extracted from cardiac tissues as well as H9c2 cells utilizing the AG RNAex Pro Reagent (Accurate Biology, China) as per the manufacturer’s instructions, followed by reverse transcription into complementary DNA using an RT reagent kit (ABclonal, USA). SYBR Green PCR Master Mix Kit (ABclonal, USA) was utilized to quantify TLR4, atrial natriuretic peptide (ANP), brain natriuretic peptide (BNP), β-major histocompatibility complex (β-MHC), CollagenI, CollagenIII, connective tissue growth factor (CTGF), and β-actin mRNA levels. The reactions were performed on a real-time PCR equipment (Bioer Technology, China) with β-actin as the internal control. The primer sequences are depicted below.

TLR4 forward 5′-CCAGAGCCGTTGGTGTATCT-3′ and reverse 5′-AGAGCATTGTCCTCCCACTC-3′.

BNP forward 5′-AGTCCTAGCCAGTCTCCAGA-3′ and reverse 5′-ATCCGGTCTATCTTGTGCCC-3′.

ANP forward 5′-GCCGGTAGAAGATGAGGTCA-3′ and reverse 5′-AGCTGGATCTTCGTAGGCTC-3′.

β-MHC forward 5′-GCAGATCATCAAGGCCAAGG-3′ and reverse 5′-AGTTGCCTCTTGAGGTCCTC-3′.

Collagen forward 5′-ATGTGCCACTCTGACTGGAA-3′ and reverse 5′-TCCATCGGTCATGCTCTCTC-3′.

CollagenIII forward 5′-CCCCTGGTTCTTCTGGACAT-3′ and reverse 5′-TGGGCCTTTGATACCTGGAG-3′.

CTGF forward 5′-GTGAGTCCTTCCAAAGCAGC-3′, reverse 5′-TAGTTGGGTCTGGGCCAAAT-3′.

β-Actin forward 5′-GGATTCCTATGTGGGCGACGA-3′ and reverse 5′-GCGTACAGGGATAGCACAGC-3′.

### 2.10 Western blot

To extract total proteins, rat heart tissues, as well as H9c2 cells, were lysed utilizing an ice-cold radio-immunoprecipitation lysis buffer (Absin, China). A BCA detection kit (Absin, China) was employed to quantify the concentration of isolated proteins. The proteins were separated by performing 10% sodium dodecyl sulfate-polyacrylamide gel electrophoresis and subsequently transferred onto polyvinylidene difluoride (PVDF) membranes using an electric transfer method. After the blocking step with a 5% nonfat dry milk solution for 1.5 h, the protein samples on the PVDF membrane were then incubated with the primary antibodies overnight at 4°C. This step allows the primary antibodies to specifically bind to their target proteins on the membrane for detection in subsequent steps. The following were the primary antibodies against various target proteins: TLR4 (A5258, 1:2,000, ABclonal), collagen I (14695-1-AP, 1:2,000, Proteintech), TGF-β (21898-1-AP, 1:2,500, Proteintech), α-S-adenosylmethionine (α-SAM) (14395-1-AP, 1:6,000, Proteintech), CaMKII (12666-2-AP, 1:2,000, Proteintech), p-CaMKII (AP0255, 1:4,000, ABclonal), NLRP3 (27458-1-AP, 1:1,000, Proteintech), gasdermin D (GSDMD) (20770-1-AP, 1:5,000, Proteintech), the N-terminal domain of gasdermin D (GSDMD-N) (ab215203, 1:1,000, Abcam), caspase-1 (22915-1-AP, 1:6,000, Proteintech), ASC (16087-1-AP, 1:3,000, Proteintech), and β-actin (81115-1-RR, 1:20,000, Proteintech). After conventional washing procedures, the PVDF membranes loaded with protein samples were incubated in HRP-conjugated secondary antibody (S0001, 1:3,000, Affinity) for 1.5 h at room temperature and visualized using an ECL buffer (Tanon, China). Image Lab software was applied to measure the strip absorbance values, which were normalized to the intensity of the β-actin band as the loading control [31].

### 2.11 Statistical analysis

Experimental data were analyzed and interpreted via utilizing GraphPad Prism 8. The results were presented in the format of mean ± standard deviation. The statistical analysis was carried out using either Student’s *t*-test or one-way analysis of variance by Tukey’s multiple comparison test. A *p*-value below 0.05 was deemed statistically significant.

## 3 Results

### 3.1 TLR4 was up-regulated in the hearts of DM rats and H9c2 cells exposed to HG

**Figure 1:**
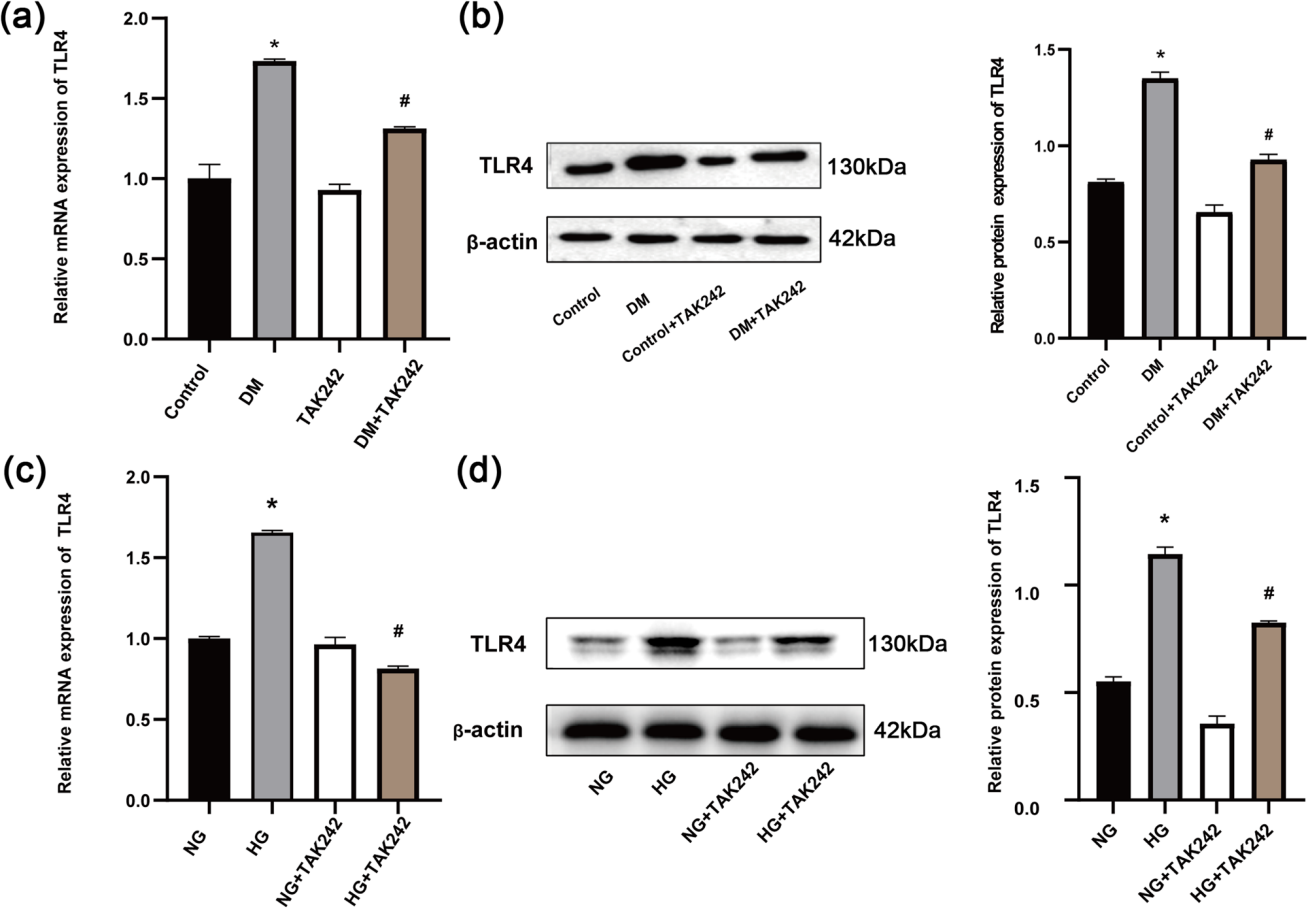
TLR4 was up-regulated in the hearts of DM rats and H9c2 cells exposed to HG. (a) TLR4 mRNA levels in the heart. (b) Protein band images and statistical analysis demonstrating TLR4 expression in the heart tissues of DM rats. (c) TLR4 mRNA levels in H9c2 cells. (d) Representative protein band pictures and statistical analysis demonstrating TLR4 expression in H9c2 cells. The *in vivo* experiment was repeated for eight times independently, and the *in vitro* for three times. *p < 0.05 versus (vs) control; ^#^ *p* < 0.05 vs DM.

We first investigated the expression of TLR4 mRNA and protein levels in the hearts of DM rats and H9c2 cells exposed to HG. The results revealed a significant elevation of TLR4 expression in the heart tissues following STZ-induced DM, as illustrated in Figure 1a and b. Furthermore, a significant elevation in TLR4 levels was observed in H9c2 cells after HG stimulation (Figure 1c and d). TAK-242 is a specific inhibitor of TLR4. In both *in vitro* and *in vivo* experiments, the treatment of TAK-242 alone did not significantly alter the expression of TLR4, which was consistent with the previous report [32]. However, when TAK-242 was administered in conjunction with DM or HG induction, it significantly inhibited the expression of TLR4 at mRNA and protein levels (Figure 1a–d).

### 3.2 STZ-induced heart injury was ameliorated for inhibition of TLR4

**Figure 2:**
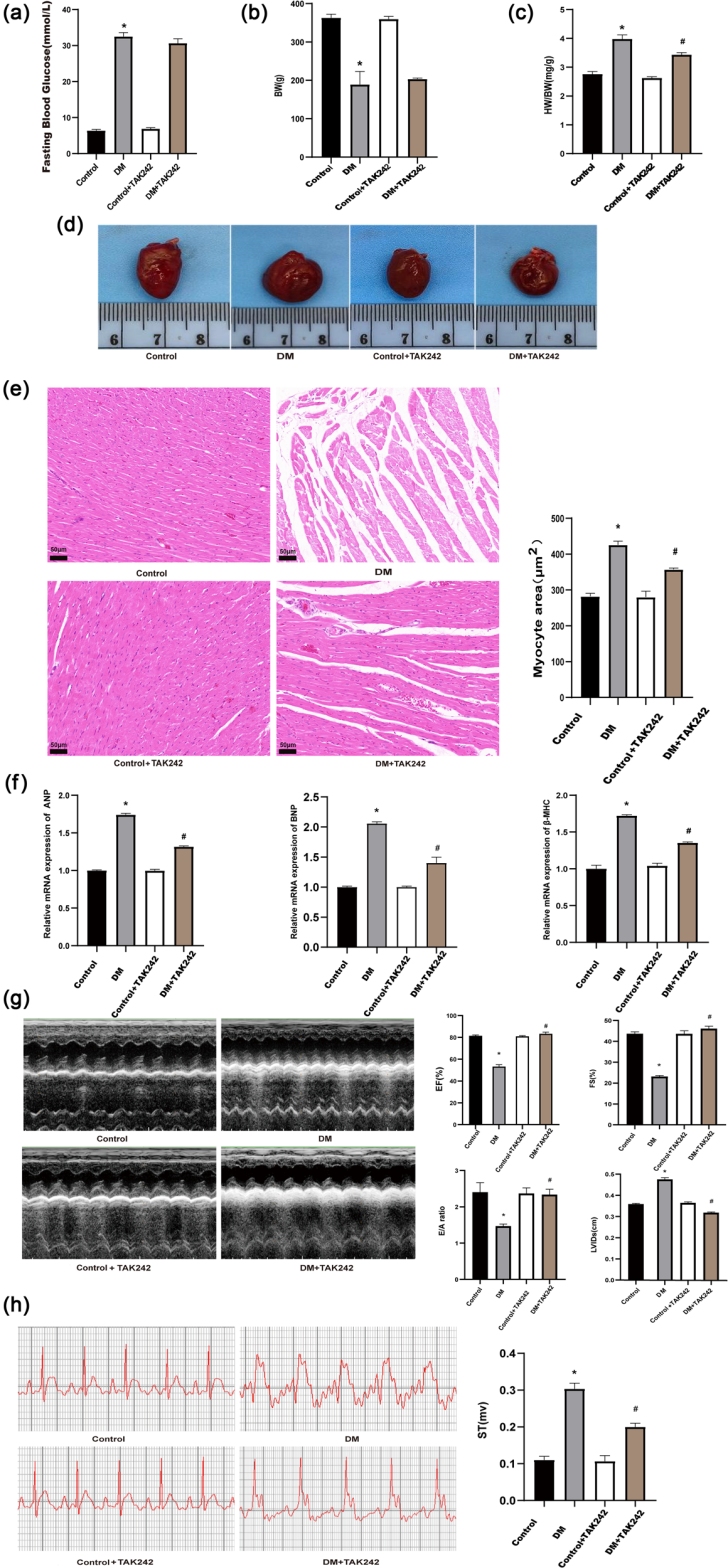
STZ-induced heart injury was ameliorated for inhibition of TLR4. FBG (a), BW (b), HW/BW (c), and morphology (d) of the rat hearts. (e) Representative photographs of heart H&E staining from various groups, as well as measurement of cardiomyocyte area. (f) qRT-PCR to detect the mRNA levels of the hypertrophic makers, including ANP, BNP, and β-MHC. (g) Echocardiographic indicator results. (h) TAK-242 effects on STZ-induced EGC alterations in rats. The *in vivo* experiment was repeated for eight times independently. *p < 0.05 vs control; ^#^ *p* < 0.05 vs DM.

DCM is one of the most prevalent consequences of diabetes, frequently resulting in substantial morbidity and mortality [33]. STZ-induced heart damage is commonly associated with the development of cardiac hypertrophy and dysfunction [34]. As depicted in Figure 2a–c, the DM group demonstrated markedly elevated FBG levels and a heightened heart weight-to-body weight (HW/BW) ratio compared to the control group, suggesting the presence of diabetes-induced cardiac hypertrophy. Treatment with TAK-242 resulted in a lower HW/BW ratio when compared to the DM group. However, no statistically significant differences were observed in FBG or BW. These findings suggested that TAK-242 therapy improved heart hypertrophy in rats with diabetes.

To further confirm the effect of TLR4 suppression on heart enlargement caused by STZ injection, heart size was measured (Figure 2d). Furthermore, myocyte area, as determined by H&E staining, revealed that TLR4 suppression played a critical role in reducing STZ-induced cardiac injury (Figure 2e). Additionally, mRNA levels of cardiac hypertrophy markers (ANP, BNP, and β-MHC) were significantly elevated in the DM group compared to the control group. TLR4 suppression with TAK-242 effectively prevented these elevations in comparison to the DM group (Figure 2f).

Moreover, cardiac function was measured utilizing the echocardiography. The results indicated that diabetes led to a notable decrease in EF, FS, and the E/A ratio, along with an increase in LVIDs compared to the control group. However, TLR4 inhibition with TAK-242 significantly improved EF, FS, and E/A ratio, while reducing LVIDs (Figure 2g). The ECG data in Figure 2h depicted that the DM group had a considerably higher ST-segment elevation compared to the control group. TAK-242 therapy clearly ameliorated the ECG abnormalities, demonstrating the protective effect of TLR4 inhibition.

### 3.3 TAK-242 attenuated fibrosis in DCM

**Figure 3:**
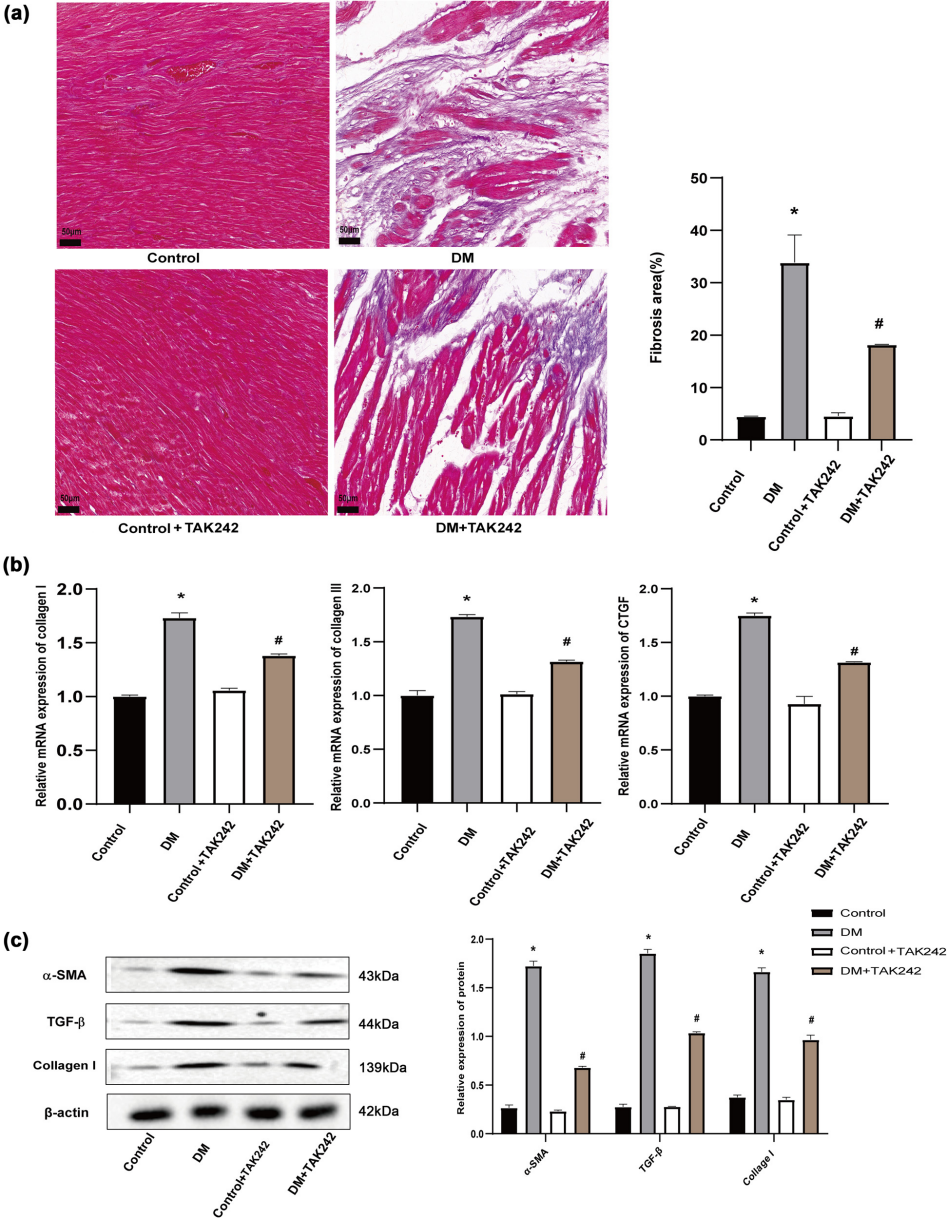
TAK-242 attenuated fibrosis in DCM. (a) Representative images of Masson trichromatic staining, as well as a quantitative analysis of the fibrotic area ratio. (b) Relative mRNA expression levels of Collagen I, III, and CTGF. (c) Quantification of collagen I, α-SMA, and TGF-β protein levels was demonstrated using Western blot. The *in vivo* experiment was repeated for eight times independently. *p < 0.05 vs control and ^#^ *p* < 0.05 vs DM.

In the context of STZ-induced DCM, DM rats exhibited higher levels of fibrosis when compared to the control group. Notably, TAK-242 treatment showed a reduction in the extent of fibrotic tissues (Figure 3a). Cardiac tissues from DM rats indicated a significant upregulation of Collagen I, Collagen III, and CTGF compared to the control. However, when comparing DM+TAK-242 to DM alone, there was a considerable downregulation of these genes (Figure 3b). Similarly, increased levels of Collagen I, α-SAM, and TGF-β proteins were observed following STZ induction, which subsequently decreased upon TAK-242 treatment (Figure 3c). These findings suggested that the attenuation of cardiac damage by TAK-242 was associated with the suppression of fibrosis.

### 3.4 TAK-242 inhibited HG-induced ROS generation *in vitro*

**Figure 4:**
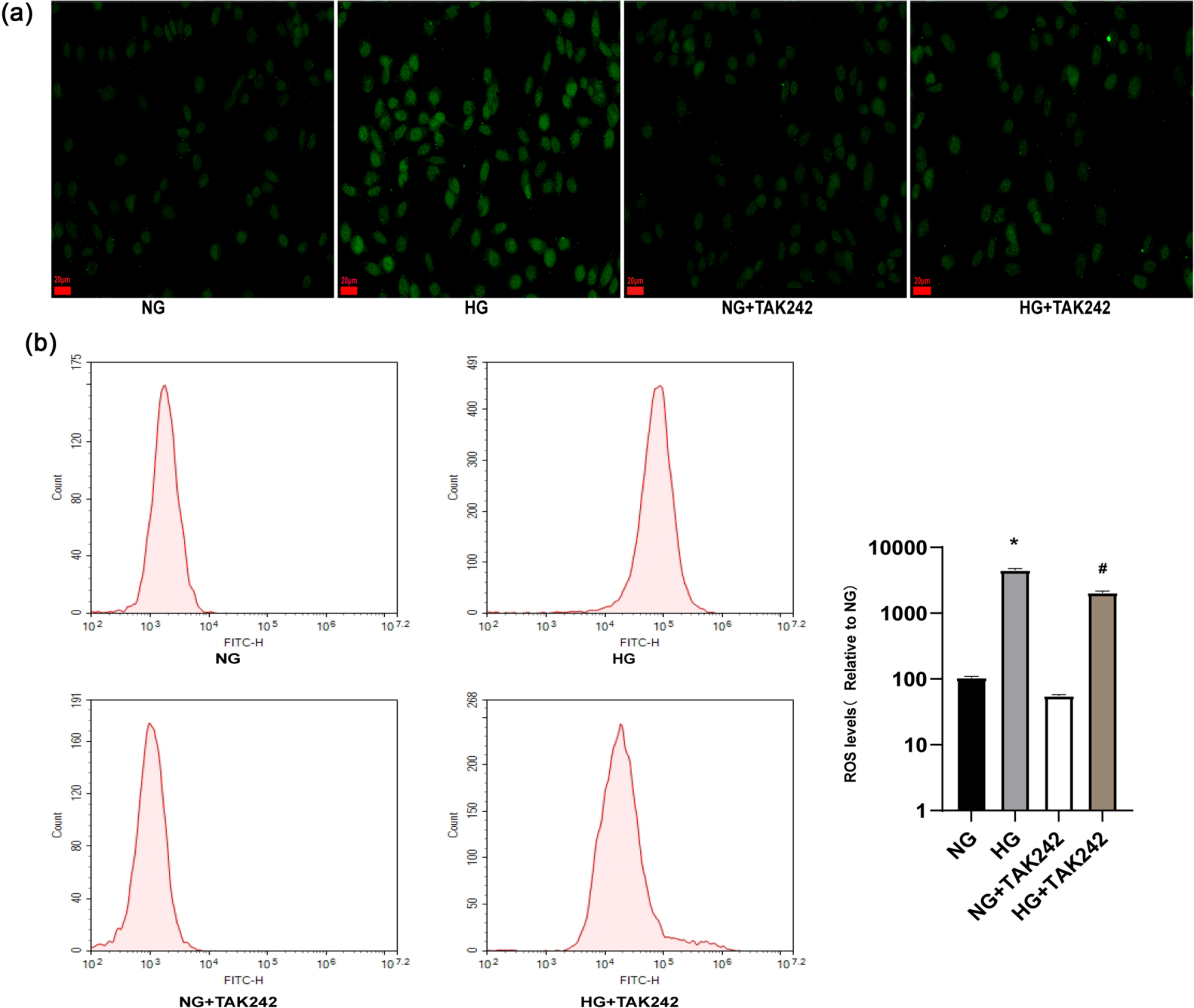
TAK-242 inhibited HG-induced ROS generation *in vitro*. DCFH-DA staining (a), flow cytometry (b) were employed to access the intracellular ROS levels. The *in vitro* experiment was repeated for three times independently. *p < 0.05 vs NG; ^#^ *p* < 0.05 vs HG.

To examine the potential antioxidative impact of TAK-242 therapy *in vitro*, DCFH-DA fluorescence was utilized to monitor ROS formation in H9c2 cells. Subsequently, confocal microscopy and flow cytometry were then employed to measure intracellular ROS levels. Our results showed that HG treatment significantly augmented ROS production in H9c2 cells. However, pretreatment with TAK-242 exhibited a remarkable reduction in HG-induced ROS production in H9c2 cells (Figure 4a and b).

### 3.5 TAK-242 inhibited CaMKII phosphorylation and cardiomyocytes pyroptosis in DM rats

**Figure 5:**
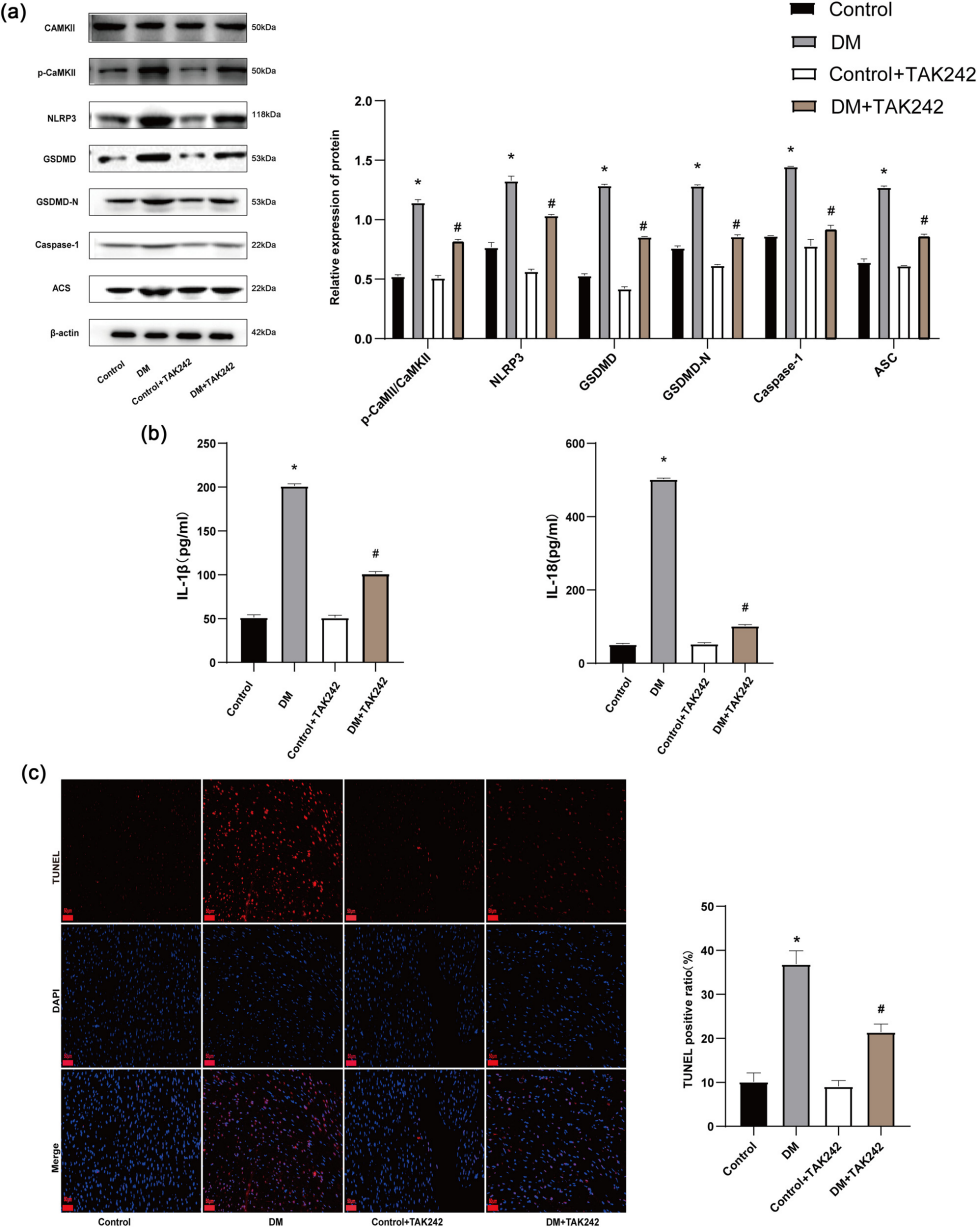
TAK-242 inhibited CaMKII phosphorylation and cardiomyocytes pyroptosis in DM rats. (a) Western blot representative images and quantitative analysis of p-CaMKII/CaMKII, NLRP3, GSDMD, GSDMD-N, Caspase 1, and ASC levels. (b) TAK-242 effects on IL-1β and IL-18 levels in rat serum. (c) Illustration of TUNEL staining and examination of positive cells. The *in vivo* experiment was repeated for eight times independently. *p < 0.05 vs control and ^#^ *p* < 0.05 vs DM.

In the DM group, there was an elevated p-CaMKII/CaMKII ratio and increased expression of pyroptosis-related proteins, including NLRP3, GSDMD, GSDMD-N, caspase 1, and ASC compared to the control group. However, TAK-242 administration resulted in a reduction of the p-CaMKII/CaMKII ratio and decreased expression of NLRP3, GSDMD, GSDMD-N, caspase 1, and ASC when compared to the DM group (Figure 5a). The DM group displayed significantly elevated concentrations of IL-1β and IL-18 in the serum compared to the control group. However, TAK-242 administration considerably lowered the serum levels of these cytokines (Figure 5b). Furthermore, the experimental results revealed a minimal presence of TUNEL-positive cells in the control group. In contrast, the DM group exhibited a significant rise in the number of TUNEL-positive cells compared to the control group. Interestingly, the DM+TAK-242 group demonstrated a reduction in TUNEL-positive cells when compared to the DM group (Figure 5c).

### 3.6 KN-93 inhibited the expression of pyroptosis-related proteins in DM rats

**Figure 6:**
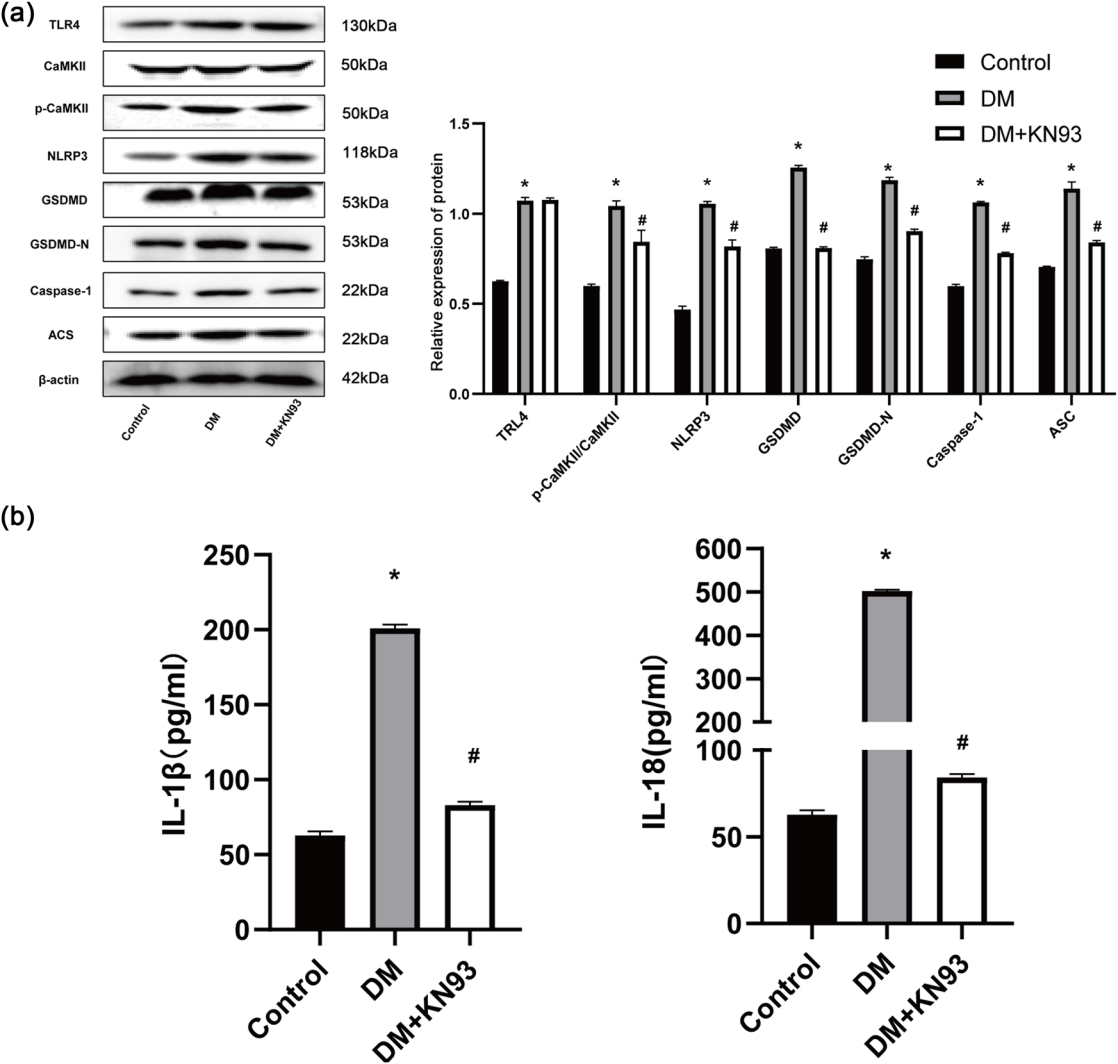
KN-93 inhibited the expression of pyroptosis-related proteins in DM rats. (a) Western blot representative images and quantitative analysis of TLR4, p-CaMKII/CaMKII, NLRP3, GSDMD, GSDMD-N, caspase 1, and ASC levels. (b) KN-93 effects on cardiac inflammatory cytokines in serum. The *in vivo* experiment was repeated for eight times independently. *p < 0.05 vs control and ^#^ *p* < 0.05 vs DM.

KN-93, a selective CaMKII inhibitor, was utilized to investigate the involvement of CaMKII in pyroptosis-related signaling. KN-93 treatment significantly decreased the p-CaMKII/CaMKII ratio as well as the protein levels of NLRP3, GSDMD, GSDMD-N, Caspase 1, and ASC when compared to the DM group. However, the intervention did not impact TLR4 expression (Figure 6a). In addition, the levels of pro-inflammatory variables such as IL-1β and IL-18 in blood were considerably lower in the DM+KN-93 group compared to the DM group (Figure 6b).

### 3.7 TAK-242 suppressed HG-induced pyroptosis in H9c2 cells

**Figure 7:**
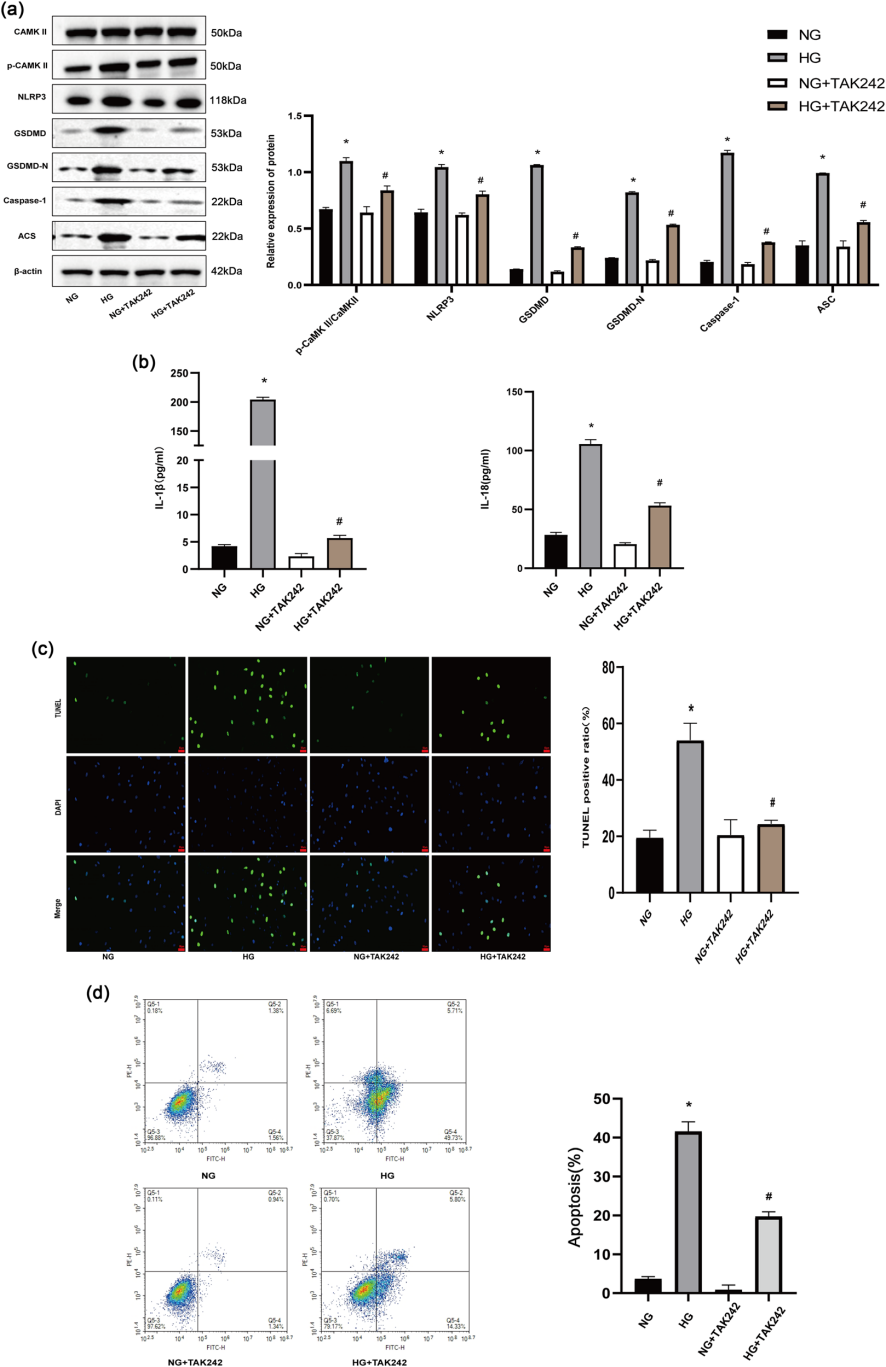
TAK-242 suppressed HG-induced pyroptosis in H9c2 cells. (a) The expression of pyroptosis pathway proteins was accessed using Western blot. (b) ELISA kits were used to detect the effect of TAK-242 on the levels of IL-1β and IL-18 in the supernatant of H9c2 cell culture media. (c) TUNEL staining assay representative images. (d) The proportion of dead cells was determined by flow cytometry. The *in vitro* experiment was repeated for three times independently. *p < 0.05 against NG and ^#^ *p* < 0.05 vs HG.

To further study the inhibitory effect of TAK-242 on HG-induced pyroptosis in H9c2 cells, the p-CaMKII/CaMKII ratio and the expression levels of NLRP3, GSDMD, GSDMD-N, Caspase 1, and ASC were measured. The results demonstrated a significant elevation of these proteins in response to HG stimulation. However, TAK-242 treatment effectively reduced the production of these key proteins, thereby inhibiting pyroptosis in H9c2 cells (Figure 7a). Moreover, HG stimulation led to a substantial increase in the production of IL-1β and IL-18 in the culture medium. However, treatment with TAK-242 effectively inhibited this rise in IL-1β and IL-18 levels (Figure 7b). In addition, incubation with HG substantially induced pyroptosis in H9c2 cells, as evidenced by an increase in TUNEL-positive cells. Conversely, TAK-242 treatment led to a decrease in TUNEL-positive cells (Figure 7c). Flow cytometry results further supported these findings, revealing an increased rate of pyroptosis in H9c2 cells following the HG challenge, which was ameliorated by TAK-242 treatment (Figure 7d). These findings suggest that TAK-242, by suppressing TLR4, attenuates HG-induced cardiomyocyte damage.

### 3.8 Inhibition of CaMKII relieved pyroptosis levels of H9c2 cells induced by HG

To confirm whether TLR4 exerted its effects via CaMKII-mediated pyroptosis, H9c2 cells were exposed to KN-93. Preliminary tests were conducted to assess the impact of TAK-242 and KN-93 on cell viability. The application of 1 mM TAK242 or 5 μM KN-93 did not result in a significant impact on cell viability. Therefore, we used 1 mM TAK242 or 5 μM KN-93 in the later experiments. In addition, we found that HG significantly reduced cell viability, while TAK-242 and KN-93 significantly alleviated HG-induced cell death (Figure S1).

**Figure 8:**
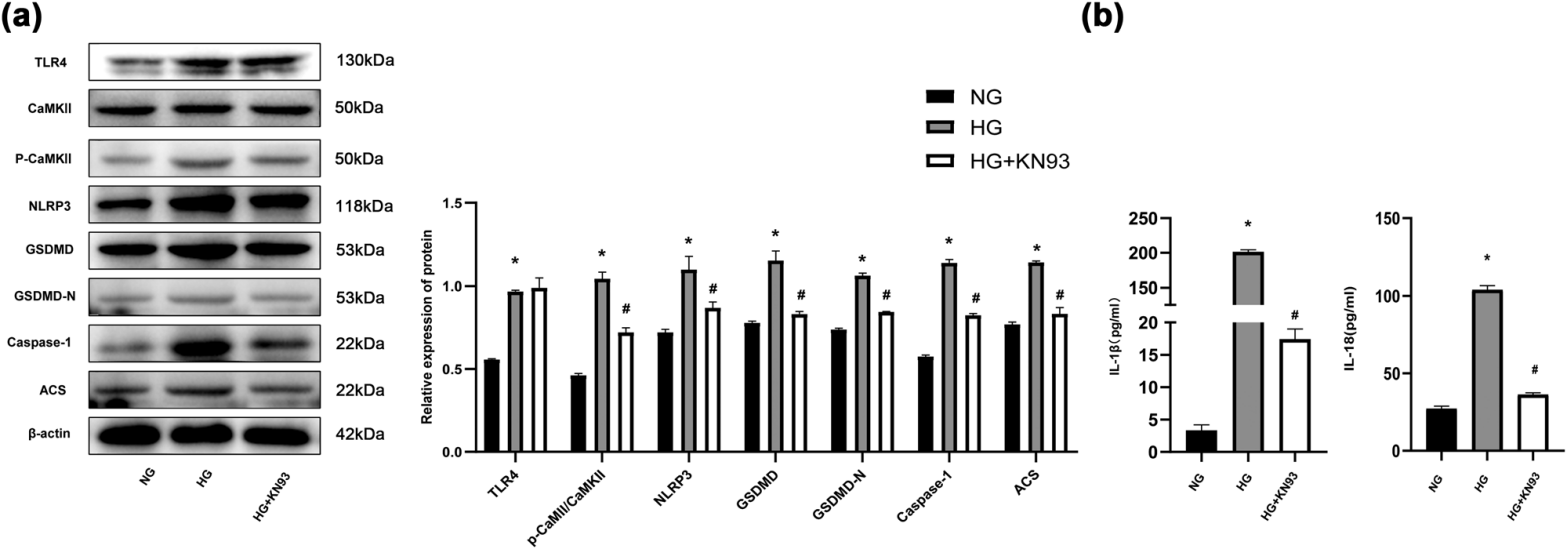
Inhibition of CaMKII relieved the pyroptosis levels of H9c2 cells induced by HG. (a) Western blot representative images and quantitative analysis of TLR4, p-CaMKII/CaMKII, NLRP3, GSDMD, GSDMD-N, caspase 1, and ASC levels. (b) ELISA was used to measure IL-1β and IL-18 levels in H9c2 cell culture supernatants. The *in vitro* experiment was repeated for three times independently. *p < 0.05 vs NG and ^#^ *p* < 0.05 vs HG.

The group given both HG and KN-93 exhibited a reduction in the enhanced levels of pyroptosis-related proteins induced by HG, while TLR4 expression remained unaffected (Figure 8a). Additionally, the ELISA analysis revealed that KN-93 significantly decreased the concentrations of IL-1β and IL-18 in the H9c2 cell culture supernatants (Figure 8b). These findings indicated that inhibiting CaMKII activation was beneficial for mitigating cell damage to some extent *in vitro*.

## 4 Discussion

Our research illustrated that targeting TLR4 possessed therapeutic potential in alleviating heart injury associated with DCM. TAK-242 treatment was effective in reducing fibrosis in DCM and inhibiting ROS production induced by HG. Furthermore, TAK-242 was able to inhibit CaMKII phosphorylation, NLRP3 expression, and pyroptosis both *in vivo* and *in vitro*. These results indicated that focusing on TLR4 and the downstream CaMKII/NLRP3-mediated pyroptosis pathway could be a promising treatment approach for DCM.

Inhibition of TLR4 could be a potential treatment target for DCM [18,19]. The previous study has shown that GTS-21 exhibited cardioprotective effects by mitigating the TLR4/NF-κB pathway in rats with STZ-induced DCM [35]. Nicotinamide mononucleotide prevented CD36-mediated lipid accumulation and CD36-TLR4 interaction, thereby reducing inflammation, fibrosis, cardiac dysfunction, and whole-body insulin resistance [36]. Our study revealed that the expression of TLR4 was elevated in the left ventricular myocardium of DCM rats. Furthermore, to evaluate the role of TLR4 in DCM, we employed TAK-242, a well-known TLR4 inhibitor, to investigate whether inhibiting TLR4 could slow disease progression. Our findings provided additional evidence that hyperglycemia contributed to hypertrophy, fibrosis, oxidative stress, pyroptosis in cardiomyocytes, and eventually leading to cardiac failure. Treatment with TAK-242 reversed cardiac hypertrophy in rats, as indicated by reduced HW/BW ratio, decreased cross-sectional area of cardiomyocytes, and downregulation of hypertrophy-related genes, such as ANP, BNP, and β-MHC. Notably, no significant change was observed in blood glucose levels or body weight between the DM+TAK-242 group and the DM group, indicating that the preventive effects of TAK-242 on DCM were not dependent on metabolic regulation.

Diabetes-induced cardiomyopathy is characterized by myocardial fibrosis as its hallmark pathology [37,38]. Previous research demonstrated that suppression of TLR4 protected experimental rats from left ventricular dysfunction and cardiac fibrosis following myocardial infarction [39]. We observed significant impairments in diastolic function, as evidenced by reduced E/A ratio, and systolic dysfunction, indicated by reduced EF and FS, in the DM group compared to the control group in our current study. Besides, treatment with TAK-242 effectively restored these functional parameters. Moreover, the inhibition of TLR4 by TAK-242 led to a significant decrease in cardiac fibrosis by downregulating the expression of the TGF-β signaling pathway. This resulted in reduced levels of fibrotic markers, including α-SMA and collagen I, in the hearts of diabetic rats. Moreover, arrhythmia, defined by elevated ST segment, is a common consequence of DCM [40,41]. Remarkably, TAK-242 treatment effectively decreased diabetic-induced ST-segment elevation, indicating its protective impact on myocardial cells.

The study unveils a novel discovery suggesting that the inhibition of the CaMKII/NLRP3 axis shows promise in suppressing pyroptosis and mitigating myocardial damage in DCM. The significant role of CaMK family members, particularly CaMKII, in disease pathogenesis and their involvement in essential cellular processes make them compelling targets for therapeutic interventions [21]. In this study, we investigated the inhibitory effect of TLR4 on CaMKII protein and delved into the underlying mechanisms. Targeting the CaMKII/NLRP3 axis has been proven to mitigate sepsis-induced cardiac injury by reducing pyroptosis [42]. TLR4 signaling has been proven to be linked to CaMKII activation, with TLR4 acting as an upstream molecule in the CaMKII signaling pathway [43]. In our experimental model of DCM induced by STZ, inhibiting TLR4 improved cardiac dysfunction and reduced the expression of pyroptosis-related proteins in myocardial tissue. Additionally, TAK-242 treatment effectively reduced the degree of cardiomyocyte pyroptosis caused by HG *in vitro*. Importantly, TAK-242-mediated suppression of TLR4 led to a reduction in p-CaMKII expression, whereas KN-93 treatment had no effect on TLR4 expression. These results provide evidence that TLR4 functions as an upstream regulator of CaMKII activity in heart damage induced by HG. Hence, our findings revealed that TAK-242 inhibited CaMKII activity, thereby preventing CaMKII-mediated cardiomyocyte pyroptosis.

The prior investigation has presented evidence implicating the NLRP3 inflammasome, which comprises NLRP3, pro-caspase 1, and apoptosis-associated speck-like protein with a CARD domain (ASC), as an inflammatory mediator in the development of DCM [44]. Following NLRP3 inflammasome activation, active caspase-1 enhances its activity in maturing the progenitors of the inflammatory cytokines IL-1β and IL-18 [45,46]. Blocking TLR4 signaling has been confirmed to decrease NLRP3 expression via decreasing the expression of nuclear factor-kappa B (NF-κB) in HG-induced cardiac cells [47]. In our study, we discovered that HG dramatically stimulated the creation and activation of NLRP3 inflammasomes, which boosted the production of mature IL-1β and IL-18 *in vitro* and *in vivo*. TAK-242 effectively reversed the pyroptosis-related alterations in NLRP3, caspase 1, IL-1β, and IL-18 in diabetic rats and HG-exposed H9c2, thereby mitigating myocardial damage.

Furthermore, TAK242 was reported to reduce ROS accumulation in H9c2 cells [48], hence decreasing HG-induced cell damage. It is worth noting that there is a close relationship between ROS generation and the pyroptosis process. Intracellular oxidative stress and increased ROS generation promote pyroptosis via the NLRP3 inflammasome [49]. The interplay between ROS and pyroptosis aggravates the inflammation, which leads to significant cell damage. However, this investigation does not provide evidence regarding the specific mechanism by which ROS influences cardiac pyroptosis. Future work is needed to investigate the role of ROS in modulating cardiomyocyte pyroptosis.

This study has several limitations that should be acknowledged. First, ethical considerations prevented the assessment of TLR4 expression in the left ventricular tissue of diabetic patients. Thus, the direct relevance of our findings to human DCM remains to be determined. Second, our study primarily utilized TAK-242 to confirm its effect and did not employ genetically modified rats specifically targeting TLR4. Future studies should consider addressing these limitations to further investigate the mechanisms underlying DCM and identify potential therapeutic interventions.

## 5 Conclusion

In summary, this study demonstrated the significance of TLR4 in STZ-induced cardiac injury and explored the impact and underlying mechanism of TAK-242 on DCM *in vitro* and *in vivo*.

Notably, this research provides novel evidence demonstrating that TAK-242, a specific TLR4 inhibitor, effectively alleviates cardiomyocyte pyroptosis by inhibiting active CaMKII and subsequently restricting the activation of pyroptosis-related proteins. These findings imply that modulating TLR4 to target the CaMKII/NLRP3 signaling pathway could be a possible treatment option for DCM.

## Supplementary Material

Supplementary Figure
