# Supplementary material for: TAK-242 alleviates diabetic cardiomyopathy via inhibiting pyroptosis and TLR4/CaMKII/NLRP3 pathway
Source: Open Life Sci. 2024 Sep 10;19(1):20220957. doi: 10.1515/biol-2022-0957 (PMC11406225; doi:10.1515/biol-2022-0957)
Supplement: Supplementary Figure [file biol-2022-0957-sm.pdf]

Supplementary material

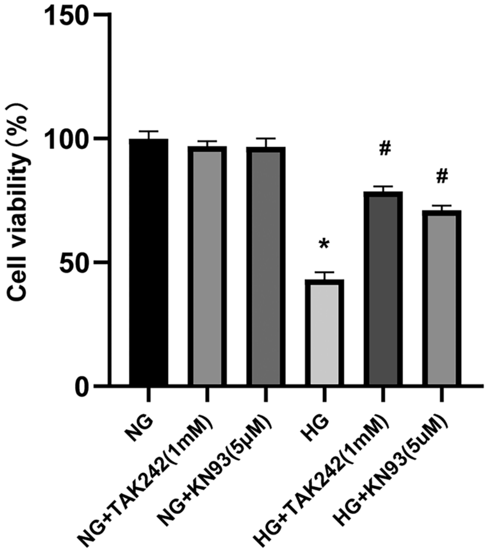

Figure S1: The effects of TAK242 and KN-93 on the proliferation of H9c2 cells were determined by CCK-8. The *in vitro* experiment was repeated for three times independently. *$p < 0.05$ vs NG and #$p < 0.05$ vs HG.
